# Obesity and diabetes are major risk factors for epicardial adipose tissue inflammation

**DOI:** 10.1172/jci.insight.145495

**Published:** 2021-08-23

**Authors:** Vishal Vyas, Hazel Blythe, Elizabeth G. Wood, Balraj Sandhar, Shah-Jalal Sarker, Damian Balmforth, Shirish G. Ambekar, John Yap, Stephen J. Edmondson, Carmelo Di Salvo, Kit Wong, Neil Roberts, Rakesh Uppal, Ben Adams, Alex Shipolini, Aung Y. Oo, David Lawrence, Shyam Kolvekar, Kulvinder S. Lall, Malcolm C. Finlay, M. Paula Longhi

**Affiliations:** 1 William Harvey Research Institute, Barts and The London School of Medicine and Dentistry, Queen Mary University of London, London, United Kingdom.; 2 Department of Cardiology, Barts Heart Centre, St Bartholomew’s Hospital, London, United Kingdom.; 3 Research Department of Medical Education, UCL Medical School, University College London, London, United Kingdom.; 4 Department of Cardiac Surgery, Barts Heart Centre, St Bartholomew’s Hospital, London, United Kingdom.; 5 University College London Hospitals NHS Foundation Trust, London, United Kingdom.

**Keywords:** Cardiology, Inflammation, Cardiovascular disease, Obesity, T cells

## Abstract

**BACKGROUND:**

Epicardial adipose tissue (EAT) directly overlies the myocardium, with changes in its morphology and volume associated with myriad cardiovascular and metabolic diseases. However, EAT’s immune structure and cellular characterization remain incompletely described. We aimed to define the immune phenotype of EAT in humans and compare such profiles across lean, obese, and diabetic patients.

**METHODS:**

We recruited 152 patients undergoing open-chest coronary artery bypass grafting (CABG), valve repair/replacement (VR) surgery, or combined CABG/VR. Patients’ clinical and biochemical data and EAT, subcutaneous adipose tissue (SAT), and preoperative blood samples were collected. Immune cell profiling was evaluated by flow cytometry and complemented by gene expression studies of immune mediators. Bulk RNA-Seq was performed in EAT across metabolic profiles to assess whole-transcriptome changes observed in lean, obese, and diabetic groups.

**RESULTS:**

Flow cytometry analysis demonstrated EAT was highly enriched in adaptive immune (T and B) cells. Although overweight/obese and diabetic patients had similar EAT cellular profiles to lean control patients, the EAT exhibited significantly (*P* ≤ 0.01) raised expression of immune mediators, including IL-1, IL-6, TNF-α, and IFN-γ. These changes were not observed in SAT or blood. Neither underlying coronary artery disease nor the presence of hypertension significantly altered the immune profiles observed. Bulk RNA-Seq demonstrated significant alterations in metabolic and inflammatory pathways in the EAT of overweight/obese patients compared with lean controls.

**CONCLUSION:**

Adaptive immune cells are the predominant immune cell constituent in human EAT and SAT. The presence of underlying cardiometabolic conditions, specifically obesity and diabetes, rather than cardiac disease phenotype appears to alter the inflammatory profile of EAT. Obese states markedly alter EAT metabolic and inflammatory signaling genes, underlining the impact of obesity on the EAT transcriptome profile.

**FUNDING:**

Barts Charity MGU0413, Abbott, Medical Research Council MR/T008059/1, and British Heart Foundation FS/13/49/30421 and PG/16/79/32419.

## Graphical abstract

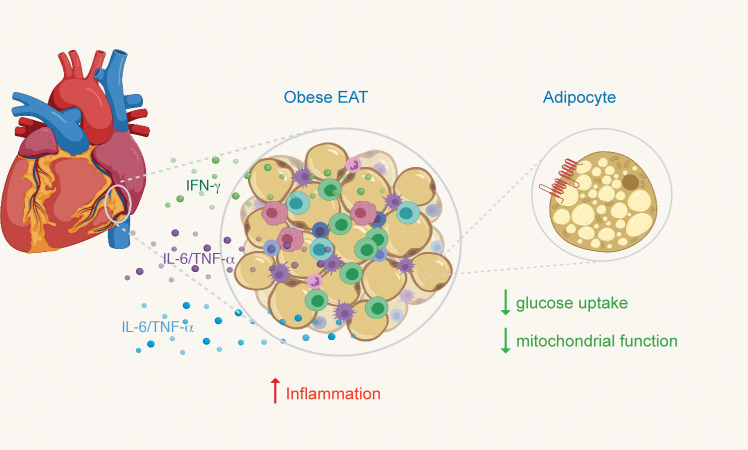

## Introduction

Epicardial adipose tissue (EAT) is the visceral adipose tissue of the heart, covering 80% of its surface and up to 20% of its weight (1). EAT is in direct contact with the underlying myocardium without fascial interruption, allowing direct interactions between the 2 tissues. EAT has been implicated in a host of cardiovascular diseases (2) and has been proposed as a transducer (3) of the effects of systemic conditions such as obesity on the heart. Significant associations between EAT thickness and cardiovascular risk factors have been reported, including diabetes (4) and hypertension (5). Although variable (4, 6, 7), a relationship has been described between EAT size and the severity of coronary artery disease (CAD) (8) as well as cardiovascular events (9, 10). However, EAT size does not necessarily correlate with the degree of inflammation observed (3), and how EAT inflammation correlates with the severity of CAD remains controversial (11).

The pivotal role of inflammation in the pathogenesis of many cardiovascular disorders is increasingly recognized (12, 13). The EAT has been identified as a rich local source of vasoactive molecules, pro- and antiinflammatory adipokines, growth factors, and other agents that can exert paracrine and vasocrine effects on the myocardium (1, 8). Inflammatory mediators released by EAT may directly contribute to the inflammation of the myocardium and coronary arteries. Indeed, higher levels of the proinflammatory mediators IL-1β, IL-6, monocyte chemoattractant protein 1 (MCP1), and TNF-α and reduced levels of adiponectin were observed in the EAT of CAD and diabetic patients (14–17). Additionally, increased infiltration of “proinflammatory” CD11c^+^ versus “antiinflammatory” CD206^+^ macrophages was observed in the EAT of CAD patients (18). Aside from these reports, much of our current understanding of adipose tissue–mediated inflammation is derived from studies of noncardiac depots, such as abdominal visceral adipose tissue, where proinflammatory macrophages predominate (19). The description of the inflammatory cell population and pathways in EAT in humans remains incomplete, and an understanding of how these may vary between conditions such as severe CAD and valvulopathy is absent.

Here, we aimed to describe the human EAT immune infiltrate and provide comparisons with subcutaneous adipose tissue (SAT) and blood inflammatory profiles (adipose tissue and systemic comparators accessible during cardiac surgery). Significant challenges in accessing human EAT were overcome by limiting this investigation to patients undergoing cardiac surgery for 2 commonly encountered cardiovascular diseases: severe CAD and valvular heart disease without severe CAD. Further, we hypothesized that the underlying risk factors associated with the EAT, specifically obesity, diabetes, and hypertension rather than CAD per se, alter the inflammatory profile of EAT. Here, we first outline the immune profile of EAT and how it differs from SAT and blood, demonstrating that T cells and not macrophages were the predominant immune cell population in both EAT and SAT. We then show that CAD and valvular heart disease patients exhibited a similar pattern of inflammation. We go on to illustrate that obesity and diabetes drove changes in immune infiltrates and that these changes were uniquely observed in the EAT, underlining its critical significance in cardiovascular disease pathophysiology.

## Results

### T cells are the primary immune cell type present in EAT.

**Figure 1:**
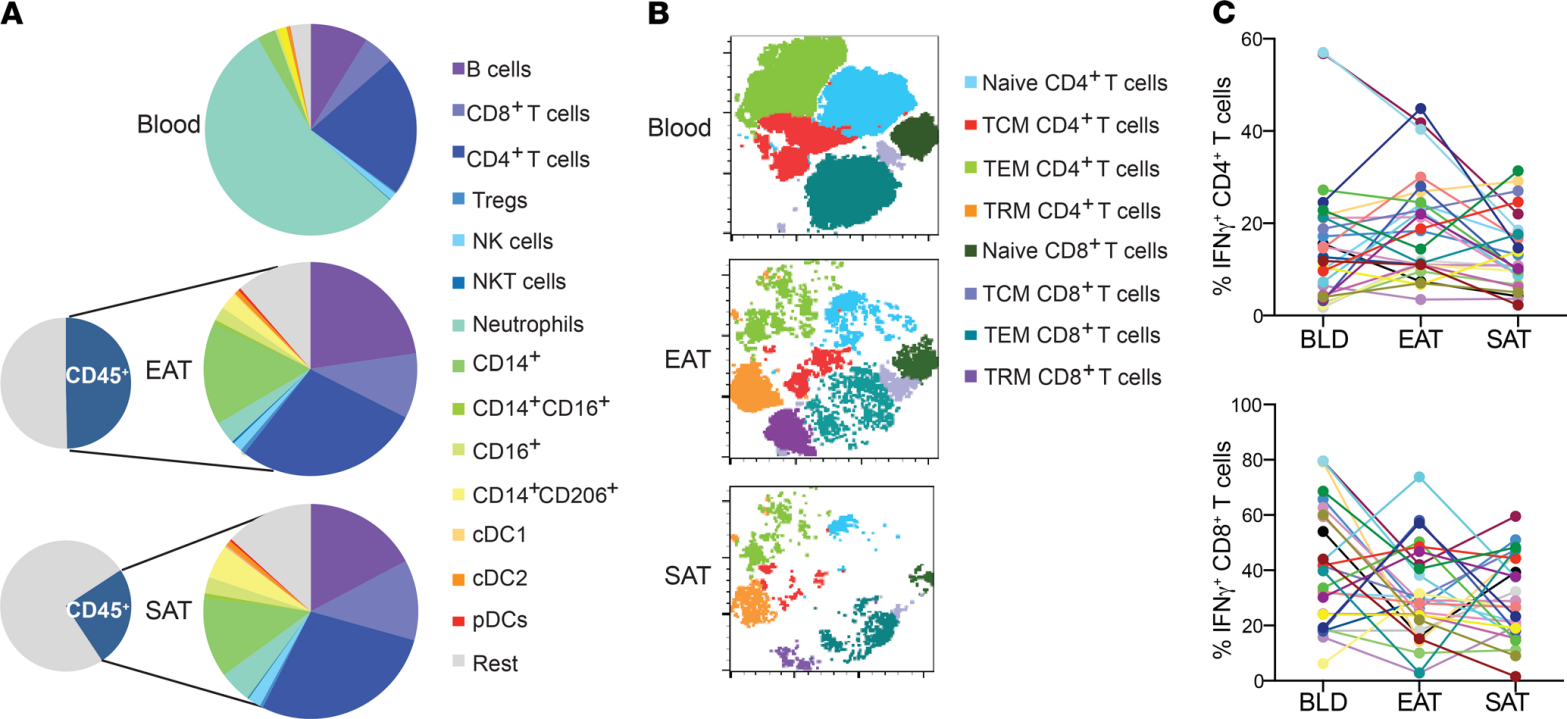
CD4+ T cells are the dominant immune population in EAT and SAT. (**A**) Pie chart illustrating relative proportions of CD45^+^ cells from the stromal-vascular fraction across tissues (*n* = 152). (**B**) Representative t-SNE plot to cluster different T cell subsets into a 2-dimensional plot across blood, EAT, and SAT. (**C**) IFN-γ levels produced by T cells in blood, EAT, and SAT (*n* = 25). Each color line represents the same patient.****

To gain a more complete understanding of the immune profile of EAT, we investigated the presence of key immune populations by flow cytometry. The gating strategy is outlined in Supplemental Figure 1; supplemental material available online with this article; https://doi.org/10.1172/jci.insight.145495DS1 The most striking differences in the immune profile were noted between blood and adipose tissue. In the blood, 60% of immune cells were neutrophils and 3% adaptive immune cells (B and T cells) while in the EAT and SAT, adaptive immune cells made up approximately 60% of immune cells and neutrophils less than 7% cells (Figure 1A). In addition, the immune infiltrate was significantly lower (*P* ≤ 0.001) in SAT than EAT. We focused particularly on T cell subsets given adipose tissue is now a well established reservoir of memory T cells (20, 21). We used unsupervised t-distributed stochastic neighbor embedding (t-SNE) plots to investigate the presence of naive T, central memory T (TCM), effector memory T (TEM), and tissue-resident memory T (TRM) cells in SAT, EAT, and blood (Figure 1B). As expected, CD4^+^ and CD8^+^ TRM cells made up a sizeable proportion of the T cell repertoire in adipose tissues but were absent in blood. TRM cells in the adipose tissue were CD103^–^, CD69^+^, and programmed cell death protein 1–positive (PD-1^+^) (Supplemental Figure 1), which was consistent with those observed in murine models (20).

Next we evaluated the phenotype of those T cells by intracellular cytokine staining in a small cohort of patients undergoing coronary artery bypass grafting (CABG) (*n* = 17) and valve repair/replacement (VR) (*n* = 8) where sufficient adipose tissue was available for additional analyses. We found that IFN-γ was the most highly expressed cytokine in both CD4^+^ and CD8^+^ T cells, with negligible levels of IL-17– and IL-22–producing T cells (Supplemental Figure 1C). However, there were marked differences in IFN-γ production in each individual patient’s EAT compared with each patient’s blood and SAT, highlighting that blood is not necessarily a good gauge of tissue-level inflammation (Figure 1C).

### EAT inflammation is independent of CAD severity.

We set out to investigate whether the immune profile of EAT from patients with severe CAD (CABG patients) differed from those with nonsevere CAD (VR patients). There were marked differences in the groups. As expected, patients were typically in their seventh decade of life; CABG and combined CABG/valve surgery patients had a high proportion of cardiovascular risk factors, including diabetes, hypertension, hyperlipidemia, and a history of smoking. Medications reflected their underlying cardiac comorbidities. Notably, baseline inflammatory markers (C-reactive protein and neutrophil/lymphocyte ratio) were similar between the groups (Table 1). In order to first comprehensively characterize the key immune populations present in the blood and adipose tissue of patients with severe versus nonsevere CAD, we used the CABG and VR surgery cohorts. Given only 16 patients had combined CABG/valve surgery, the group was too small for inferential statistical analysis following the propensity matching and hence was not included in this analysis. Patients with a history of myocardial infarction were excluded. A total of 48 patients in the CABG and VR surgery groups were thus included (Table 2) following propensity matching.

**Figure 2:**
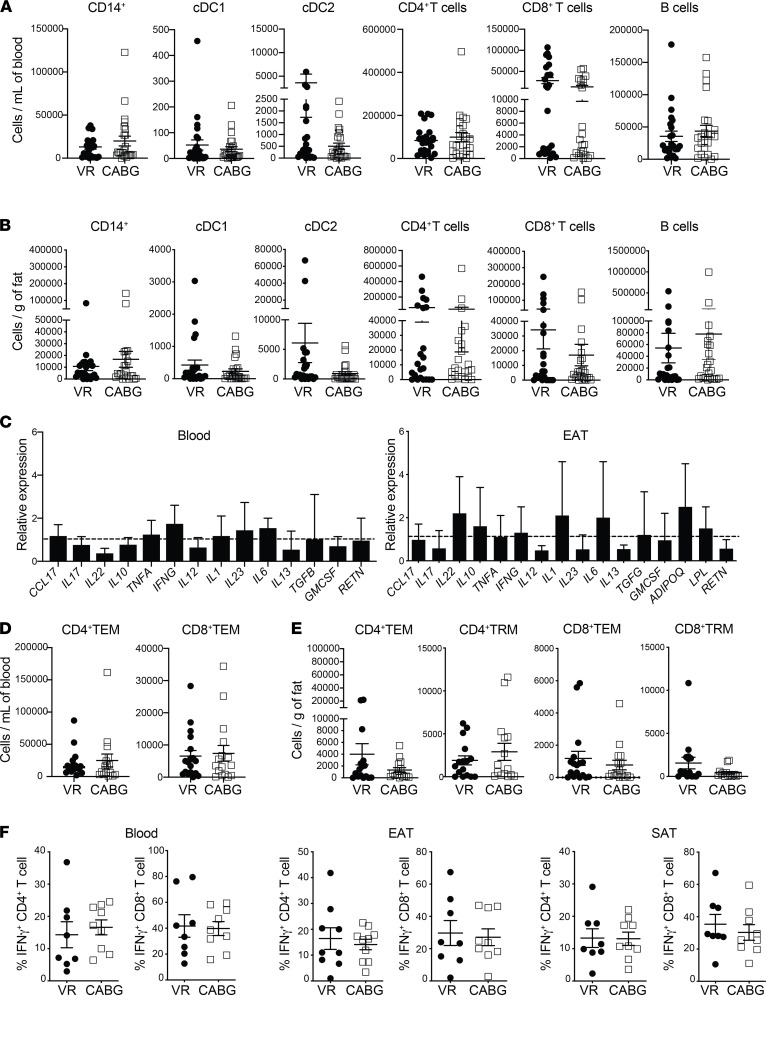
Comparison of EAT immune profiling between CAD patients and controls. (**A** and **B**) Absolute number of immune cells in CABG and VR patients across blood (**A**) and EAT (**B**) (*n* = 24 patients/group). (**C**) Relative expression levels of immune mediators in blood and EAT, respectively (*n* = 24 patients/group). Expression levels were normalized to GAPDH expression. Bars represent expression in CABG patients compared with VR surgery, which was set at 1 and indicated with dotted lines. Error bars show the geometric mean. (**D** and **E**) Graphs showing T cell subsets in blood (**D**) and EAT (**E**). (**F**) IFN-γ production among live CD4^+^ and CD8^+^ T cells in blood, EAT, and SAT, respectively (*n* = 7–8 patients/group). Statistical significance was determined by the Mann-Whitney *U* test, and data are represented as median and interquartile range.

The numbers of macrophages remained similar between groups as did the proportion of M2-polarized “antiinflammatory” CD206^+^ macrophages. Similarly, no significant differences (*P* ≤ 0.9) were noted in numbers of key immune cells in both adipose tissue and blood across both groups (Figure 2 and Supplemental Figure 2). Similarly, no differences in expression levels of immune mediators were observed across the 2 groups (Figure 2C). Reviewing cell numbers in blood and adipose tissue, again no significant changes in T cell subsets were noted, nor were there any differences in IFN-γ production across the EAT, SAT, or blood (Figure 2, D–F). Thus, our data demonstrate that CAD is not associated with alterations in immune cell numbers or inflammatory mediator levels in EAT.

### Obesity promotes EAT inflammation.

Hypercholesterolemia, overweight/obesity, hypertension, and diabetes are known to be key risk factors for the development of CAD. Thus, the next step was to identify whether these conditions are associated with an altered inflammatory picture observed in different patients.

First, we considered the effect of obesity as this is most likely to affect adipose tissue biology directly, inducing an inflammatory response. Given that patients with type 2 diabetes (T2D) were in general prescribed antihyperglycemic medications, they were analyzed as a separate group despite having similar BMIs to the overweight/obese cohort. Table 3 details the demographic and clinical characteristics of the patients utilized for the overweight/obesity and T2D group analysis.

**Figure 3:**
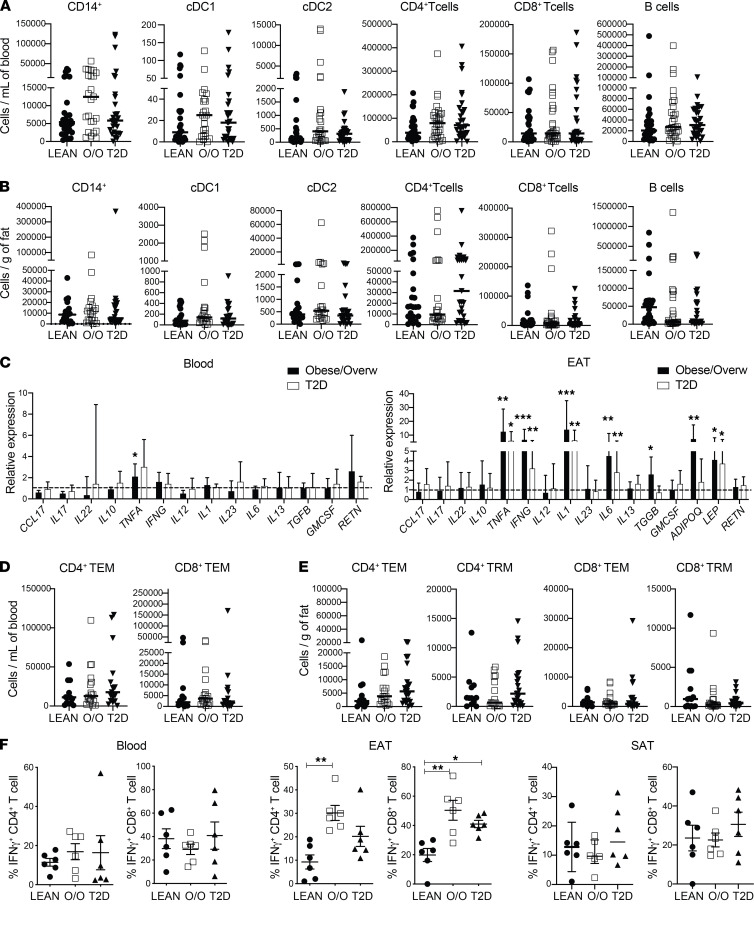
Comparison of EAT immune profiling between obese/overweight and T2D patients and controls. (**A** and **B**) Absolute number of immune cells in overweight/obesity (O/O) and T2D patients across blood (**A**) and EAT (**B**) (*n* = 30 patients/group). (**C**) Relative expression of immune mediators in overweight/obesity and T2D patients compared with lean nondiabetic patients across blood and EAT (*n* = 30 patients/group). Gene expression was normalized to GAPDH and control set as 1, indicated with dotted lines. Error bars show the geometric mean. (**D** and **E**) Graphs showing T cell subsets in blood (**D**) and (**E**) EAT. (**F**) IFN-γ production among live CD4^+^ and CD8^+^ T cells in blood, EAT, and SAT, respectively (*n* = 6 patients/group). Statistical significance was determined by the Kruskal-Wallis test with Dunn’s multiple comparisons posttest correction applied. Significance denoted as *P < 0.05, **P < 0.005, ***P < 0.0005, and data are represented as median and interquartile range.

Absolute numbers of key adaptive immune cells were similar across groups (Figure 3, A, B, D, and E; and Supplemental Figure 3). However, the immune mediator profile was dramatically altered uniquely in the EAT compared with blood and SAT (Figure 3C and Supplemental Figure 4). Overweight/obese and T2D patients were observed to have greatly elevated expression levels of the same proinflammatory immune mediators, specifically a 5- to 10-fold increase in IL-1, IL-6, TNF-α, and IFN-γ. IL-6 and TNF-α can be produced by myeloid cells and adipocytes, while IL-1 and IFN-γ are mainly released by immune cells, e.g., myeloid cells and T cells, respectively. All these cytokines are known to contribute to adipose tissue inflammation and insulin resistance. As expected, overweight/obese and T2D patients exhibited increased expression levels of the adipokine leptin. Notably, levels of adiponectin were elevated in obese patients. The proportion of TEM and TRM cells was similar between groups. Yet a clear increase in CD4^+^IFN-γ^+^ and CD8^+^IFN-γ^+^ T cells could be observed in both overweight/obese and T2D groups, seeing as T cells are the main producer of IFN-γ. The level of immune mediators in blood was similar, with the exception of TNF-α that was elevated in the overweight/obese cohort. These data indicate that although the cellular immune infiltrate in EAT remains the same, obesity and diabetes induce a change in phenotype toward a proinflammatory state.

### Obesity induces extensive EAT remodeling.

**Figure 4:**
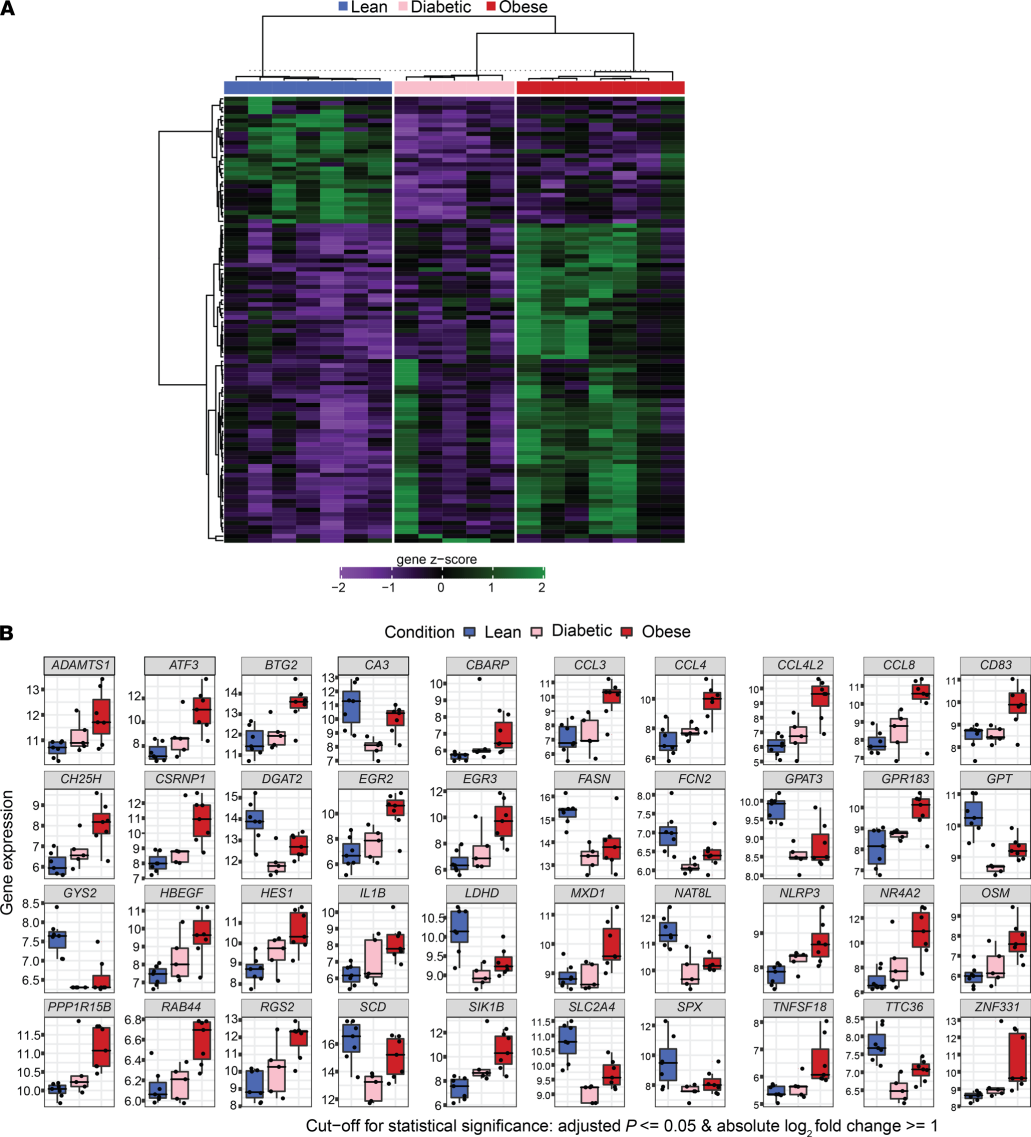
Differentially expressed genes in EAT from obese/overweight and T2D patients compared with lean. (**A**) Heatmap of differentially expressed genes in EAT, cutoff: adjusted *P* < 0.05; log_2 _fold change > 1. (**B**) Bars represent gene expression value for the top 40 differentially expressed genes in all 3 groups.

In order to further investigate the differences in gene expression in EAT between lean and overweight/obese and T2D patients, we performed bulk RNA-Seq. The thresholds of differentially expressed genes were fold change more than 2 and adjusted *P* value less than 0.05. There were 133 differentially expressed genes in overweight/obese compared with lean EAT patients, while 94 were found to be significantly upregulated in T2D patients (Figure 4A and Supplemental Figure 5A). Inflammatory and metabolic genes were differentially regulated in both overweight/obese and T2D samples compared with lean (Figure 4B and Supplemental Tables 1 and 2). Upregulation of inflammatory genes indicating activation of myeloid and lymphoid cells included *IL6*, *IL1b*, *CXCL8*, *CCL3*, *NLRP3*, *ATF3*, *OSM*, *CD83*, *GPR183*, *VCAN*, *CCR3*, *CXCL12*, and *CCL2*. T2D patients showed a lower expression of inflammatory genes in EAT compared with overweight/obese (Figure 4, A and B, and Supplemental Figure 5B). A similar trend was observed in the real-time PCR (RT-PCR) and intracellular cytokine production by T cells (Figure 3, C and F). Obesity induces a profound metabolic rewiring in EAT, with downregulation of genes associated with glucose metabolism (e.g., *SLC24A*, *CS*, *GPT*, *OGDH*, *ACO2*, *GPI*, *LDHD*) and lipid metabolism (e.g., *GYS2*, *GPAT3*, *CRAT*, *FASN*, *ACADVL*, *DGAT1*, *DGAT2*, *NAT8L*, *SCD*) as well as changes in genes related to adipogenesis (*HES1*, *MXD3*, *NR4A2*, *RGS2*, *PPP1R15B*, *ADAMTS1*, *CEBPD*, *KDM7A*). The metabolic phenotype was more evident in EAT from diabetic patients (Figure 4B and Supplemental Figure 5, C and D). Interestingly, certain genes associated with CAD were dysregulated in obese EAT, such as *HBEGF*, *ADAMTS1*, and *ADAMTS4* while the novel adipokine spexin (*SPX*), which regulates adipose tissue inflammation and was shown to protect cardiomyocytes from hypoxia-induced metabolic distress (22), was downregulated in EAT from both obese and diabetic patients. Overall, these data highlight significant metabolic and inflammatory changes in EAT that obesity induced.

### EAT inflammation is not associated with hypertension.

**Figure 5:**
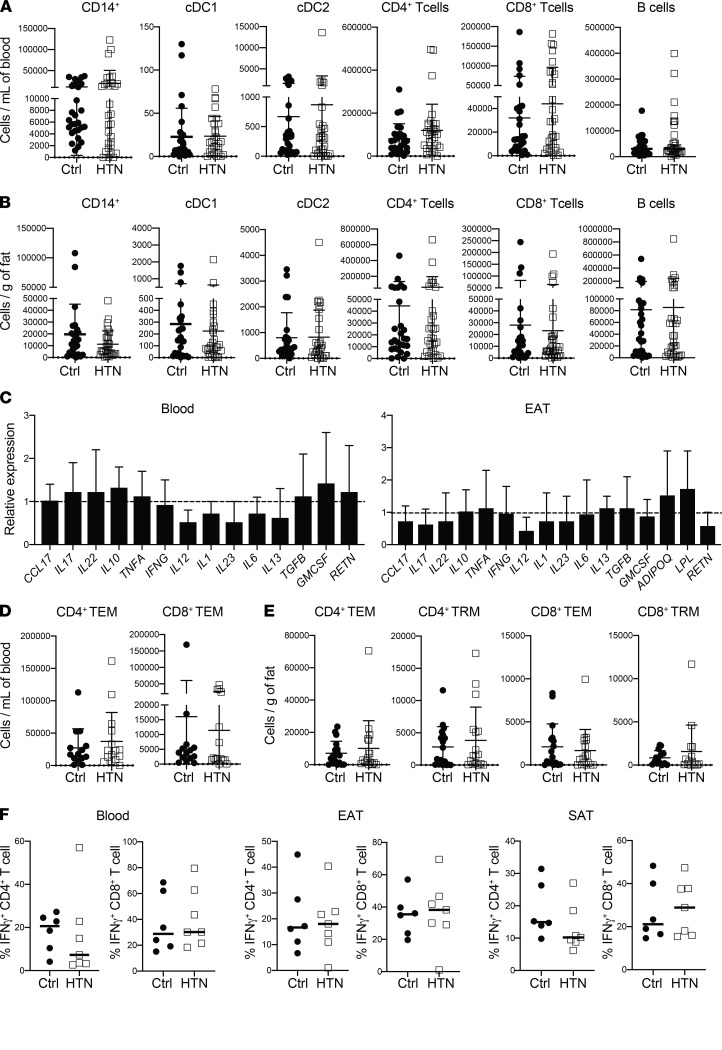
Comparison of EAT immune profiling between hypertensive patients and controls. (**A** and **B**) Absolute number of immune cells in hypertensive and control patients across blood (**A**) and EAT (**B**) (*n* = 32 patients/group). (**C**) Relative expression of immune mediators in hypertensive versus control patients across blood and EAT (*n* = 32 patients/group). Gene expression was normalized to GAPDH and control set as 1, indicated with dotted lines. Error bars show the geometric mean. (**D** and **E**) Graphs showing T cell subsets in blood (**D**) and EAT (**E**). (**F**) Graphs represent IFN-γ production among live CD4^+^ and CD8^+^ T cells in blood, EAT, and SAT, respectively (*n* = 6 patients/group). Statistical significance was determined by Mann-Whitney *U* test, and data are represented as median and interquartile range.

EAT volume was found to be increased in hypertensive compared with normotensive patients (5). Thus, we investigated if the presence of hypertension alters the EAT inflammatory state (Table 4) and found that it did not (*P* ≥ 0.14). Both hypertensive and nonhypertensive (control) patients demonstrated similar immune cell profiles in the blood, EAT, and SAT (Figure 5, A and B, and Supplemental Figure 6) alongside similar relative expression levels of key immune mediators (Figure 5C). Looking at specific T cell subsets and their phenotype, again similar absolute numbers of T cell subsets and IFN-γ production were seen across both groups and across tissues (Figure 5, D–F, and Supplemental Figure 6). Hence, we show that hypertension as a cardiovascular risk factor in isolation is not a driver of EAT inflammation.

## Discussion

A number of previous analyses (11, 15) studying patients undergoing cardiac surgery with and without significant CAD (CABG vs. VR surgery patients) suggest that it is the specific adipose tissue depot, namely EAT, that drives the unique inflammatory changes observed. With EAT’s anatomical intimacy with the myocardium and lack of fascial boundaries between them, immune mediators can have a direct and potentially deleterious impact on the heart. This highlights the unique significance of EAT in giving an indication of the local tissue environment.

To date and despite a wealth of data indicating a link between EAT volume and cardiovascular disease, in-depth investigations of EAT inflammation remain sparse. Indeed, we still lack a detailed overview of the immune profile of EAT and how it differs between SAT and blood samples and in different cardiovascular conditions. Here we show a clear enrichment of adaptive immune cells, in particular CD4^+^ T cells, in the adipose tissue (EAT and SAT) compared with blood, where neutrophils are the dominant cell type. Of note, much of the earlier literature has focused on innate immune cells, such as macrophages within EAT (16, 18), yet the immune profiling illustrates these represent a lower proportion of the immune (CD45^+^) cell population compared with adaptive immune cells. Both EAT and abdominal adipose tissue are derived from the splanchnopleuric mesoderm (1), and both tissues utilize the same vasculature and lymph drainage as their underlying organs, the intestine and myocardium, respectively. However, we have demonstrated EAT is dominated by adaptive immune cells but abdominal adipose tissue by macrophages (23). This would suggest that it is the anatomical location of the adipose tissue that determines its immune profile.

The notable finding of TRM cells within the EAT is deserving of comment. These have not previously been characterized in EAT to our knowledge. As the name indicates, TRM cells remain local to the tissue, affording long-lasting immune surveillance, and can rapidly reactivate and recruit circulating T cells when required (24). Their presence enables adipose tissue to act as a local source of adaptive immune cells; such organ-specific immunity has been previously described in other organs (25). Notably, TRM cells are high in PD-1 expression; TRM cell reactivation has been implicated in numerous cancers, but the subset’s exact role in the heart remains enigmatic (26).

We have highlighted that blood and SAT may offer a comparative lack of insight into the local tissue environment, given the dominant immune mediator produced from T cells (IFN-γ) can vary considerably among tissues in a single individual. This is a critical point as blood is typically assayed for its accessibility in giving an indication of the patient’s condition and inflammatory status, yet blood may bear little correlation with the local tissue inflammation. Indeed, this point was highlighted in one of the earliest descriptions of EAT inflammatory mediators (14). Clinicians should be mindful of the limitations in insights available from blood analysis alone.

A key finding is that once underlying cardiovascular risk factors are balanced, we have demonstrated no differences in the inflammatory profile between severe versus nonsevere CAD. Some previous publications (15, 27, 28) have reported the EAT of patients with significant CAD to have a unique inflammatory profile. However, it is noteworthy that many of these reports failed to control for other important cardiometabolic conditions, such as diabetes and overweight/obesity (16–18), or those predominantly affecting lean individuals (28), which are not typical of patients undergoing cardiac surgery. A significant strength of our work is that the large patient cohort allowed propensity matching to be performed, reducing bias between our patient groups. Our extensive comparison of a range of immune cells and immune mediators shows the immune profile to be similar in patients with severe (CABG) and nonsevere (VR) CAD across blood, EAT, and SAT. Indeed, no differences in absolute numbers of immune cells were observed in overweight/obese patients or those with diabetes. However, the immune mediators are greatly elevated in both overweight/obesity and diabetes compared with lean patients. In EAT, TNF-α, IFN-γ, IL-1, IL-6, and leptin levels were all elevated several-fold in both of these groups. Inflammation is a key mechanism of cardiovascular disease. Proinflammatory cytokines, such as IL-1 and TNF-α, can amplify local inflammation, regulate endothelial cell activation, and induce ROS production and cardiomyocyte apoptosis. Bulk RNA-Seq transcriptomic analysis further confirmed this finding. Proinflammatory cytokines were elevated in overweight/obese EAT and to a lesser extent in EAT from T2D patients. Pathway enrichment analysis further indicated upregulation of pathways associated with adaptive immune responses in addition to monocyte/macrophage activation. A reduced inflammatory response in T2D patients could be attributed to the antidiabetic drug metformin, which is considered to have an antiinflammatory effect (29). The overwhelming majority of T2D patients in our cohort were on metformin, given it is a first-line indicated drug for T2D patients. The study lacked statistical power to evaluate the effect of metformin in EAT inflammation. Cytokine-targeting therapies have emerged as possible noninvasive treatments for heart disease. Considering that adipose tissue is the primary organ affected by overnutrition and given the anatomical proximity among EAT, coronary arteries, and myocardium, it is possible to envisage EAT as the primary source of heart inflammation in obesity.

T cells were found to be the main producers of IFN-γ. Certain mediators have previously been described to be elevated in the EAT (15, 27) but not attributed to specific comorbidities through a robust propensity-matched analysis. Similarly, a host of studies have illustrated increased EAT size in patients with increased BMI (4, 6) and diabetes (15, 27), but a detailed understanding of changes in immune cell types and inflammatory mediators has been lacking. Importantly, we have demonstrated that adiponectin was uniquely elevated in the overweight/obese group. Reduced adiponectin in EAT was found to be associated with atherosclerotic plaque development, but this association is not consistent between studies (16, 18, 30), with reports not accounting for potential confounding variables, such as BMI (16), and with patients being entirely or almost exclusively men (16, 31). Adiposity and diabetes appear to alter specific inflammatory mediators rather than having a more holistic impact on the immune phenotype. This is crucial for future studies analyzing immune differences of adipose tissue between patients with varying metabolic risk profiles.

Infiltration of inflammatory cells in expanding adipose tissue can result in adipocyte dysfunction and metabolic dysregulation. Several human studies, mainly in subcutaneous fat, have linked obesity to reduced mitochondrial oxidative metabolism and biogenesis as well as to impaired glucose and lipid metabolism in adipose tissue (32, 33). The insulin-regulated glucose receptor GLUT4 is downregulated in obese adipose tissue but not skeletal muscle; however, deletion of GLUT4 selectively in adipose tissue is sufficient to induce insulin resistance (34). Downregulation of GLUT4 results in reduced adipocyte glucose uptake and de novo fatty acid synthesis (35), contributing to systemic metabolic perturbations in obesity. Our findings in EAT are in agreement with previous studies showing decreased glucose metabolism and lipid synthesis in obese adipose tissue from other sites (abdominal and subcutaneous).

Adipose tissue is one of the main sites of mitochondrial branched-chain amino acid (BCAA; leucine, valine, and isoleucine) catabolism (36). Impaired BCAA metabolism correlates with insulin resistance, altered cardiac metabolism, and greater risk of cardiovascular disease (33, 37, 38). Genes associated with BCAA catabolism, including *ACAD8*, *ALDH6A1*, *LDH9A1*, *BCKDHA*, *BCKDHB*, and *HADH*, were downregulated in EAT from overweight/obese and T2D patients, which may contribute to cardiac dysfunction. Overall, our data suggest that the metabolic perturbation observed in obese adipose tissue from other sites is similarly observed in EAT.

Finally, EAT size has been associated with the presence of hypertension (5, 39). However, hypertension does not appear to specifically affect the EAT immune phenotype. First, this highlights a point alluded to earlier that EAT size does not necessarily correlate with the degree of inflammation. Second, when considering the studies assessing hypertension and EAT, it is noteworthy that hypertensive patients often tend to be comorbid with overweight/obesity and T2D, which when adjusted for, reduces the significance of the association with EAT size and hypertension (7, 39). This is consistent with the changes observed uniquely in the overweight/obese and T2D groups in our study, when comorbidities have been adjusted for.

A number of limitations to our study are important to recognize. The number of patients per group was relatively small compared with other observational studies, but this was the largest detailed immunophenotype analysis in EAT. In addition, the immune mediator alterations observed in the EAT of overweight/obese and diabetic patients are independent associations. A causal relationship between these conditions and EAT inflammation cannot be established given the inability to perform a randomized controlled trial with the prolonged period required for conditions such as obesity and diabetes to exert an impact on EAT. Moreover, EAT can only be ethically sampled during cardiac surgery (and not in healthy volunteers, for instance). Due to the challenges in safely harvesting EAT, we were not able to perform multisite EAT sampling to assess whether regional differences in the EAT inflammatory profile could be observed from the same patient. We utilized the CABG versus VR surgery groups to compare severe versus nonsevere CAD. While we recognize these are not ideal comparison groups, as alluded to above, healthy controls could of course not be recruited. The decision for CABG was based on a joint cardiology/cardiac surgery multidisciplinary team discussion concluding severe CAD was present, warranting CABG. VR alone was performed with a similar multidisciplinary team discussion concluding there was not severe enough CAD to require concomitant CABG and VR. Thus, VR was used as a control group accepting the limitations as outlined above. In the context of these limitations, we surmised that a propensity-matched analysis using fresh human tissue samples was the best approach to study the risk factor/EAT inflammation relationship.

In summary, we have performed a detailed immune analysis of EAT, SAT, and blood in a substantial cohort of clinically well phenotyped patients undergoing cardiac surgery. We have demonstrated the overall immune profile was dominated by adaptive immune cells in EAT and SAT compared with neutrophils in blood, and the blood was not necessarily an accurate gauge of tissue-level inflammation. Finally, we have shown that key cardiometabolic conditions, namely overweight/obesity and T2D, were independently associated with significant changes in the EAT inflammatory picture rather than just the presence or absence of severe CAD.

## Methods

### Study population and sample collection.

Adult patients (≥18 years) undergoing open-chest CABG, VR, or combined CABG/valve surgery were recruited from Barts Heart Centre, St Bartholomew’s Hospital, from 2017 to 2020 (*n* = 152). Exclusion criteria included patients with underlying congenital heart disease, those with coexisting systemic inflammatory/neoplastic disorders, and those on immunomodulatory agents. Fasting blood samples were collected preoperatively in the anesthetic room. Approximately 0.8–1 g of adipose tissue samples were collected in ice-cold phosphate-buffered saline with 2% fetal bovine serum. SAT was collected immediately following the median sternotomy incision, and EAT was obtained following opening up of the pericardial sac with tissue typically collected over the body of the right ventricle. Following tissue collection, samples were transferred to the William Harvey Research Institute, Queen Mary University of London, for further processing (Supplemental Figure 7).

The protocols of the studies complied with the Declaration of Helsinki, and all patients provided informed written consent. The demographic characteristics are presented in Tables 1–4.

### Sample processing.

Fasting blood samples were collected preoperatively to include 6.5 mL of peripheral blood divided into 2.5 mL collected in a PAXgene (PreAnalytiX) tube for RNA isolation and the remaining 4 mL in an EDTA tube (BD). PBMCs were isolated using Ficoll-Paque PLUS (GE Healthcare, now Cytiva) as per manufacturer’s instructions. PBMCs were then stained using antibodies for flow cytometry analysis (Supplemental Table 3). The gating strategy is depicted in Supplemental Figure 1.

Following adipose tissue sample collection, samples were divided into a portion for flow cytometry (~0.1–0.4 g) analysis; a portion for subsequent RNA extraction (~0.1–0.2 g), which was snap-frozen; and a sample fixed in 4% formaldehyde solution (MilliporeSigma) for future immunohistochemical analysis (~0.05–0.2 g). The sample of adipose tissue aliquoted for flow cytometry analysis was first mechanically minced using microscopy scissors and then digested enzymatically using 5668 IU collagenase II (MilliporeSigma) and 55.5 IU DNase (MilliporeSigma) per gram of adipose tissue for 30 minutes. Immune cells present in the stromal vascular fraction were obtained following centrifugation and lysed for red blood cells prior to antibody staining.

### T cell stimulation assays.

In a proportion of patients where sufficient adipose tissue samples were available for further analyses, tissue was aliquoted for T cell stimulation assays. Briefly, PMA/ionomycin with the addition of brefeldin A was used to stimulate the immune cell fraction of the digested adipose tissue for 4 hours followed by IL-17, IL-22, and IFN-γ intracellular staining as shown in Supplemental Figure 1.

### RT-PCR analysis.

Total RNA was extracted from the adipose tissue using QIAzol (QIAGEN) and the RNeasy Lipid Tissue Mini Kits (QIAGEN) following the manufacturer’s instructions. Total RNA was extracted from whole blood samples stored in PAXgene (PreAnalytiX) tubes using the PAXgene blood RNA kit (QIAGEN). RNA was quantified using the NanoDrop spectrophotometer (Thermo Fisher Scientific). Reverse transcription to cDNA was performed using High-Capacity RNA-to-cDNA kits (Applied Biosystems, Thermo Fisher Scientific) and stored at –80°C. The relevant primer sequences can be found in Supplemental Table 4 and were purchased from Invitrogen, Thermo Fisher Scientific. Gene expression was performed using SYBR Green Supermix (Bio-Rad), as per manufacturer’s instructions, and analyzed using the Light Cycler System (Roche). Relative gene expression values were determined using the ΔΔCT method and normalized to a stable reference housekeeping gene control (GAPDH). The control values were set at 1. Given the ΔΔCT method is not normally distributed, the geometric mean was used for the representation of the data (40). Illumina sequencing was carried out at Novogene Bioinformatics Technology Ltd. Data were deposited in the National Center for Biotechnology Information’s Gene Expression Omnibus with accession number GSE179455.

### Propensity matching of groups.

To account for differences in baseline clinical variables between groups, a propensity matching algorithm was applied. A 1:1 propensity score matching (PSM) optimal algorithm was utilized using dedicated propensity matching software (XLSTAT, Addisoft). The confidence interval was set at 95% and a caliper width at 0.2. For the initial analysis comparing differences in severe versus nonsevere CAD, CABG and VR surgery were used as the dependent variables and age, sex, BMI, hypertension, hyperlipidemia, and diabetes as covariates. For the PSM of subsequent group analyses, BMI and diabetes were used as dependent variables and age, sex, hypertension, and hyperlipidemia as covariates. For the hypertension group analysis, the variables to be matched for included age, sex, BMI, hyperlipidemia, and diabetes status.

### Role of comorbidities and cardiovascular risk factors in subgroup assessment.

We evaluated cardiovascular risk factors that are known to impact adipose tissue specifically, namely obesity/overweight (4, 6), diabetes (15, 27), and hypertension (5, 39), to determine the extent to which they alter the inflammatory profile in different groups of patients. All diabetic patients included had T2D.

Flow cytometry analysis, RT-PCR analysis, and T cell stimulation assays (as per the above methodology) were undertaken to identify the key immune cells and mediators that differed between the groups. Comprehensive absolute changes in numbers of all immune cells are available in Supplemental Figures 3–5.

### Statistics.

Power calculations were based an effect size of 1.16, power of 0.95, and α error of 0.05. Statistical significance was determined for continuous variables where 3 groups were assessed using the 2-way ANOVA (or Kruskal-Wallis for nonparametric data) test with Dunnett’s T3 (or Dunn’s for nonparametric data) multiple comparisons posttest applied. The 2-tailed Student’s *t* test was used for 2 groups of continuous data where the data were parametric and the Mann-Whitney *U* test for nonparametric data. The χ^2^ test or Fisher’s exact test was utilized for categorical data. Data were analyzed on GraphPad Prism version 8 (GraphPad Software LLC). Normality was assessed using the Kolmogorov-Smirnov and Shapiro-Wilk tests. Where parametric data are represented, the mean and standard deviation values are reported, and for nonparametric data, median and interquartile ranges are reported. A *P* value of less than 0.05 was considered statistically significant.

### Study approval.

The study was approved institutionally via the ethical framework of the Barts BioResource (research ethics committee reference: 14/EE/0007; London, United Kingdom), and written informed consent was obtained from each patient. Written consent was obtained prior to sample collection, and patients were identified with an arbitrary Barts BioResource number.

## Author contributions

MPL and MCF conceived the study; VV, HB, EGW, and MPL designed experiments; VV, HB, EGW, and BS performed and analyzed experiments; SJS provided statistical analysis; and DB, SGA, JY, SJE, CDS, KW, NR RU, BA, AS, AYO, DL, SK, and KSL provided clinical assessment and tissue samples. VV and MPL wrote the manuscript.

Among the co–first authors, VV was assigned first given he wrote the manuscript with MPL, followed by HB and then EGW based on their relative contribution to performing and analyzing experiments.

## Supplementary Material

Supplemental data

## Figures and Tables

**Table 4:**
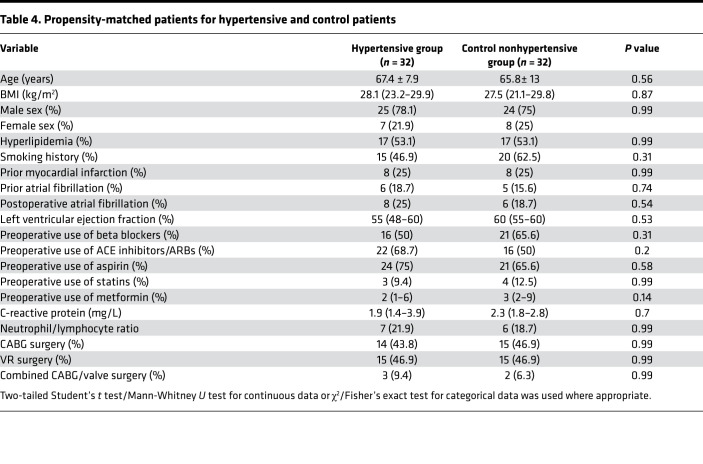
Propensity-matched patients for hypertensive and control patients

**Table 3:**
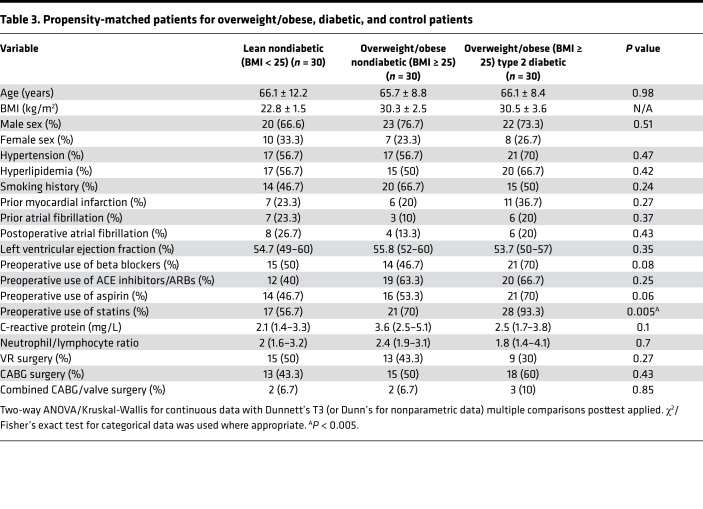
Propensity-matched patients for overweight/obese, diabetic, and control patients

**Table 1:**
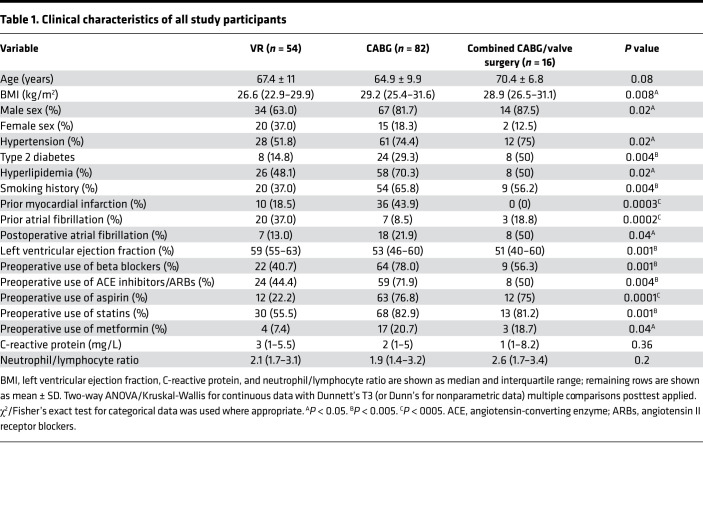
Clinical characteristics of all study participants

**Table 2:**
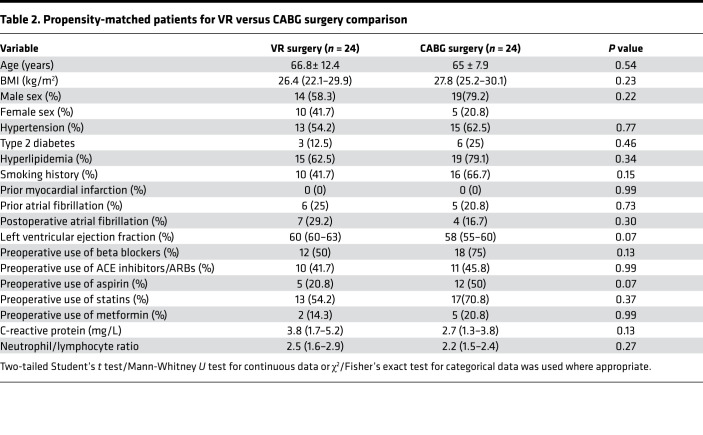
Propensity-matched patients for VR versus CABG surgery comparison

## Version 1 07/20/2021

In-Press Preview

## Version 2 08/23/2021

Electronic publication
