# Supplementary material for: Obesity and diabetes are major risk factors for epicardial adipose tissue inflammation
Source: JCI Insight. 2021 Aug 23;6(16):e145495. doi: 10.1172/jci.insight.145495 (PMC8409986; doi:10.1172/jci.insight.145495)
Supplement: Supplemental data [file jciinsight-6-145495-s236.pdf]

Supplementary material

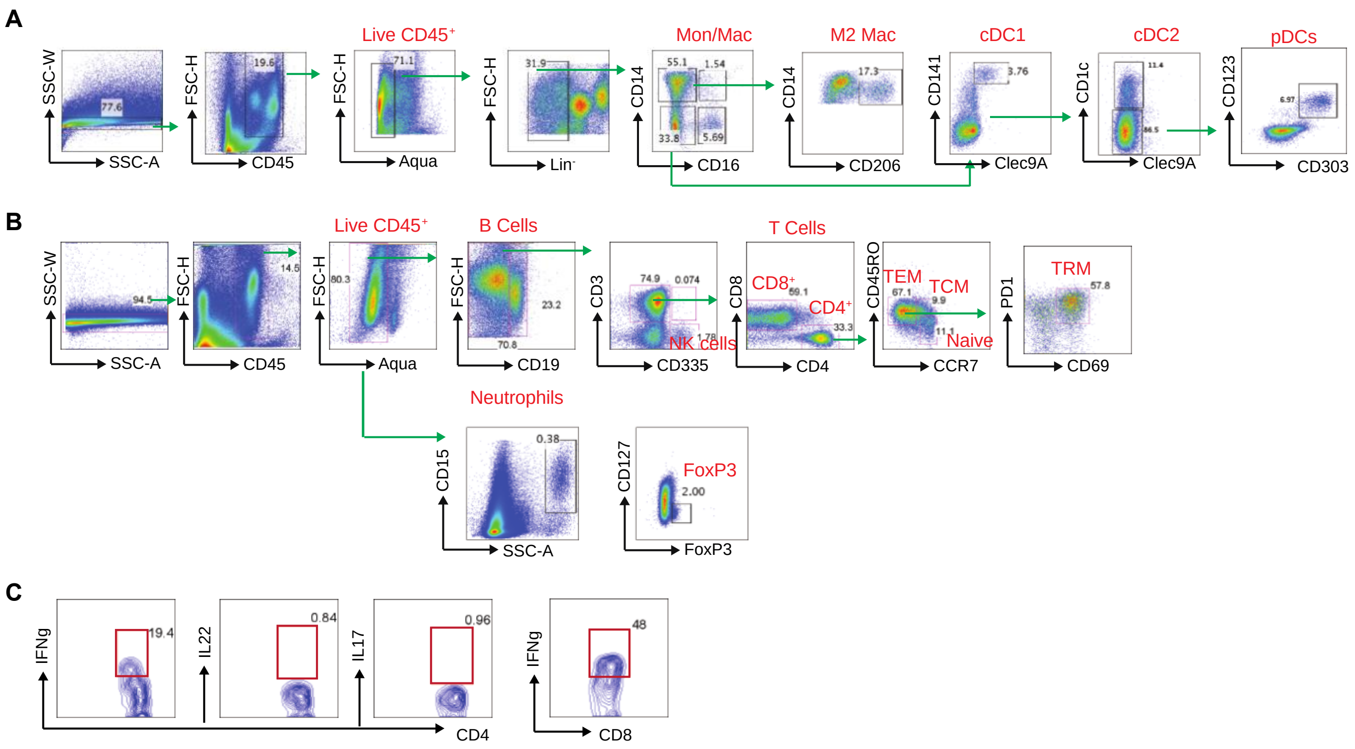

Supplementary figure 1. Flow cytometry gating strategy and immune phenotyping in blood and adipose tissue. (A) and (B) Scheme illustrating the gating used for analysis of immune cell populations in EAT. (C) Identification of proportion of cytokines produced by $CD4^+$ and $CD8^+$ T cells following T cell stimulation assays. Of note, blood samples are devoid of $CD206^+$ macrophages and TRM cells.

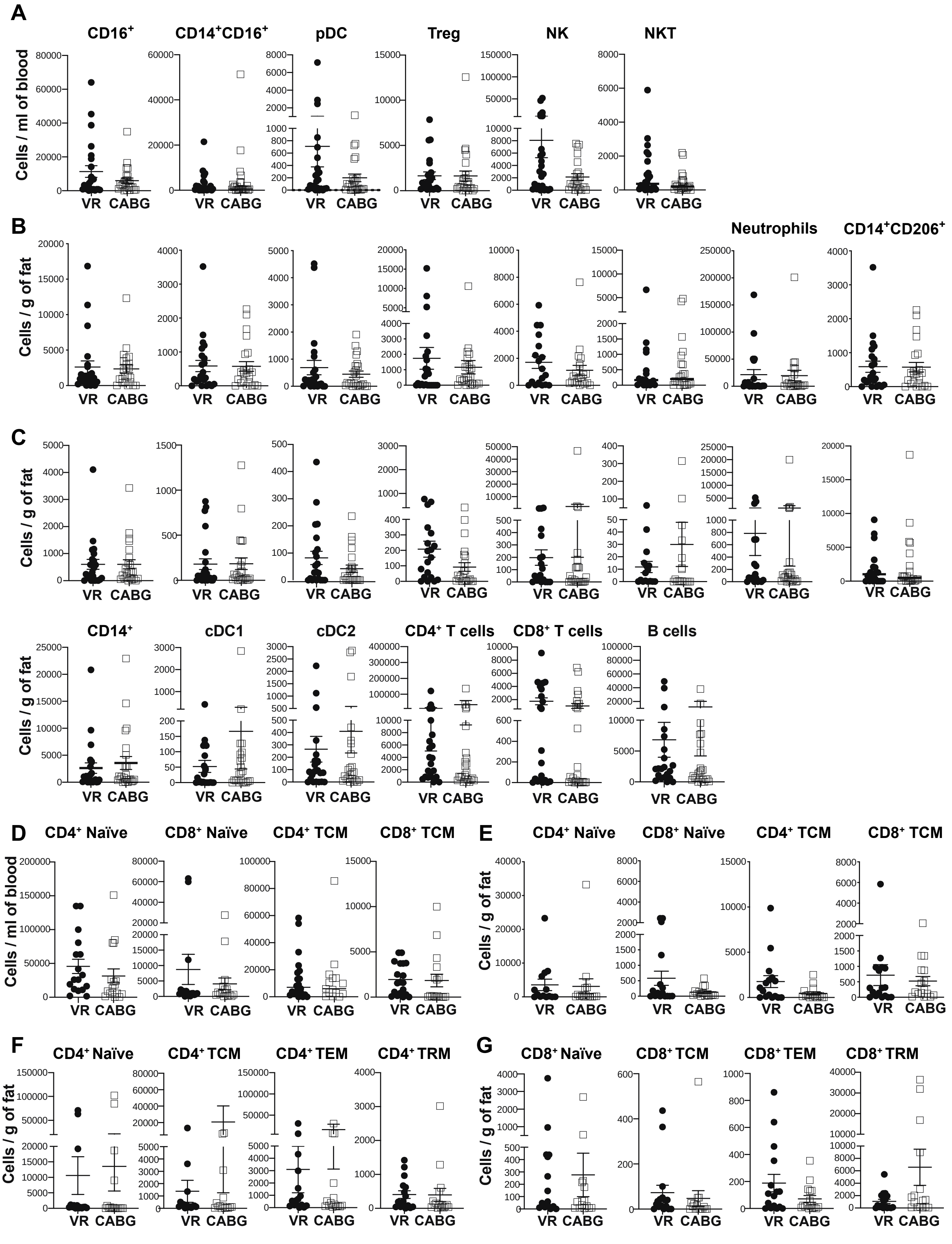

Supplementary figure 2. Absolute numbers of immune cells in blood, EAT and SAT in CAD and control patients. Graphs illustrating absolute number of a range of immune cells in CABG and VR surgery patients across blood (A), EAT (B) and SAT (C). Graphs represent T cell subset absolute numbers in blood (D), EAT (E) and SAT (F-G). Statistical significance was determined by Mann-Whitney test, and data represented as median(n=24 patients/ group).

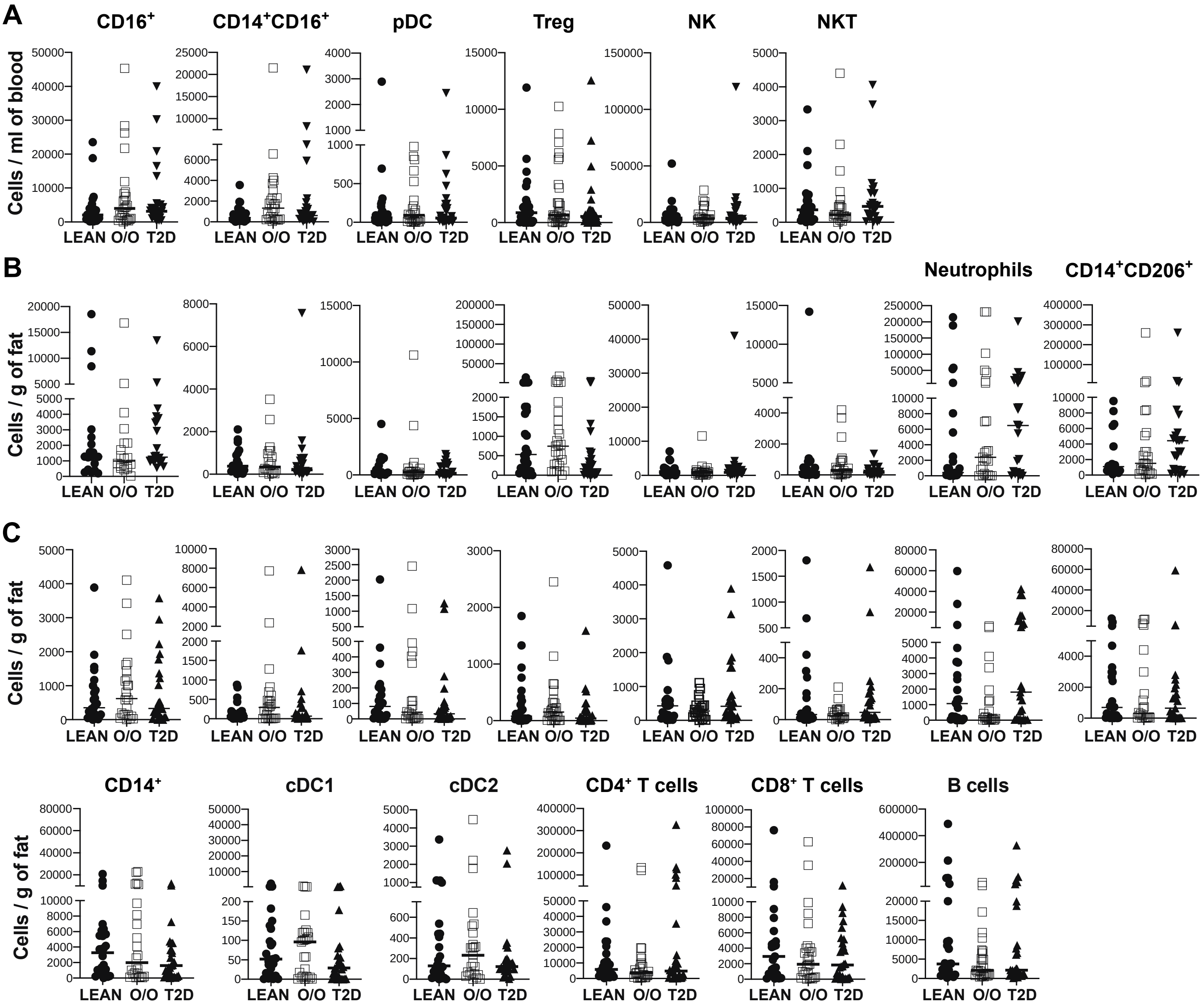

Supplementary figure 3. Absolute numbers of immune cells in blood, EAT and SAT in Obese/Overweight, T2D and control patients. Graphs illustrating absolute number of a range of immune cells in overweight/obesity and T2D patients across blood (A), EAT (B), SAT (C). Statistical significance was determined by the Kruskall-Wallis test with Dunn's multiple comparisons post-test correction applied. Data represented as median(n=30 patients/ group).

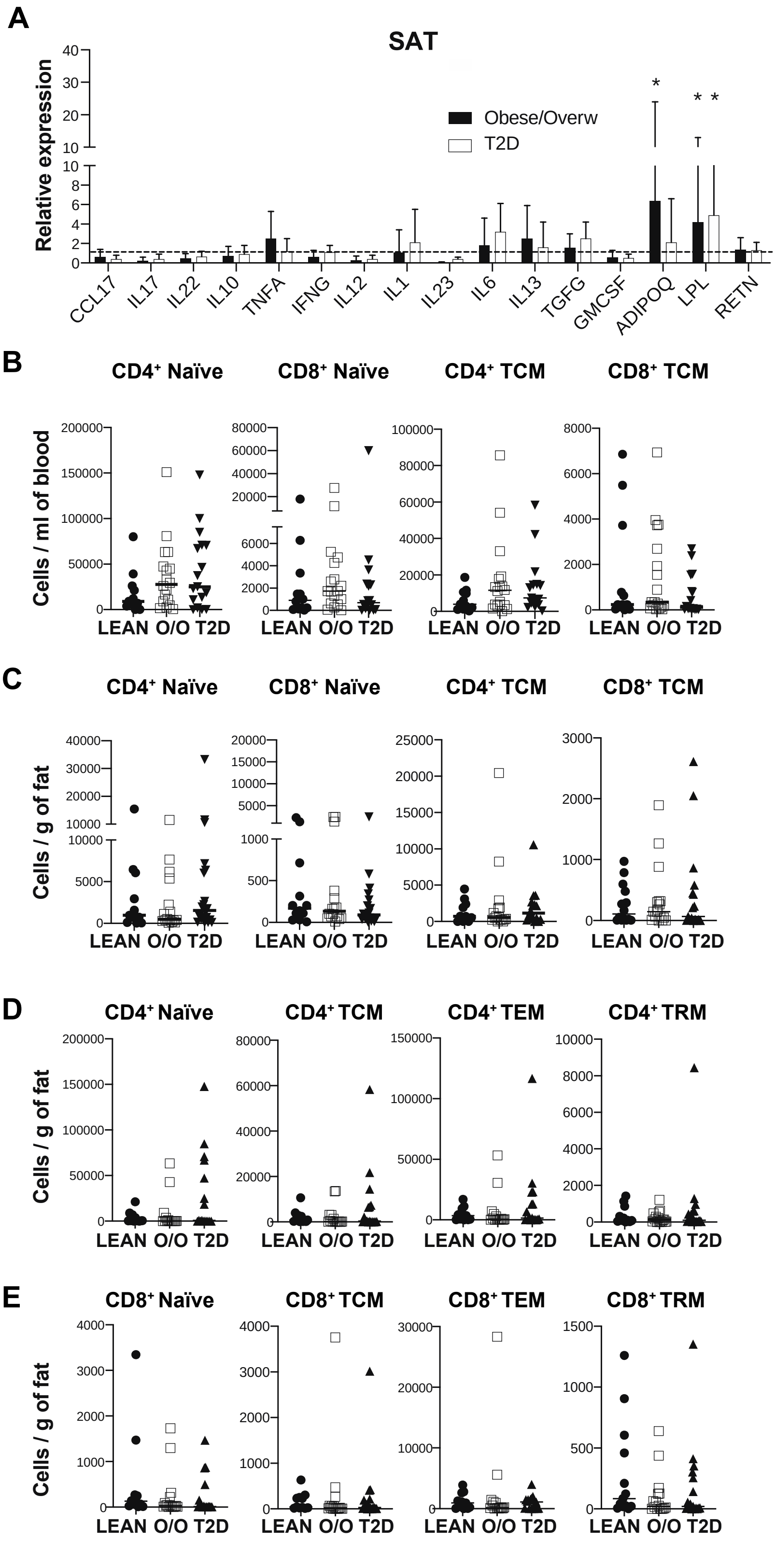

Supplementary figure 4. Absolute numbers of T cell subsets in blood, EAT and SAT in Obese/Overweight, T2D and control patients. (A) Relative expression levels of immune mediators in overweight/obesity and T2D patients compared with lean non-diabetic patients in SAT . All gene expression levels normalised against the housekeeping gene GAPDH and control set as 1, indicated with doted lines. Error bars show the geometric mean. (B-E) Graphs showing T cell subsets in blood (B), EAT (E), and SAT (D-E). Statistical significance was determined by the Kruskall-Wallis Tests with Dunn's multiple comparisons post-test correction applied. Significance denoted as *p<0.05; **p<0.005 and data represented as median(n=30 patients/ group).

A

Variable	Lean non-diabetic (BMI<25) (n=7)	Overweight /obese non-diabetic (BMI≥25) (n=7)	Overweight /obese (BMI≥25) Type 2 diabetic (n=5)	P value
Age (years)	63.4 ± 8.9	63.7 ± 8.4	65 ± 9	0.87
Body Mass Index (kg/m^2)	22.9 ± 0.9	29.82 ± 1.1	28.4 ± 2	N/A
Male Gender (%)	5 (71)	6 (85)	4 (80)	0.8
Hypertension (%)	4 (57)	3 (43)	3 (60)	0.8
Hyperlipidemia (%)	4 (57)	4 (57)	4 (80)	0.4
Smoking history (%)	3 (43)	5 (71)	4 (80)	0.35
Prior myocardial infarction (%)	3 (43)	2 (29)	2 (40)	0.84
Left ventricular ejection fraction (%)	55.2 (49-60)	50.67 (30-75)	55 (42-68)	0.67
Pre-operative use of beta blockers (%)	3 (43)	4 (57)	4 (80)	0.43
Pre-operative use of ACE inhibitors/ARBs (%)	3 (43)	5 (71)	3 (60)	0.55
Pre-operative use of Aspirin (%)	3 (43)	5 (71)	3 (60)	0.55
Pre-operative use of Statins (%)	3 (43)	5 (71)	4 (80)	0.35
CRP (mg/L)	2 (1-3)	4.4 (1.8 -7.2)	3.5 (1.2-7.7)	0.12
Neutrophil: lymphocyte ratio	4.4 (3-5.6)	5.1(3.3-7.2)	3.1 (3.1-5.3)	0.57
Valve repair/replacement surgery (%)	3 (43)	2 (29)	2 (40)	0.34
CABG surgery (%)	4 (57)	5 (71)	3 (60)	0.34

B

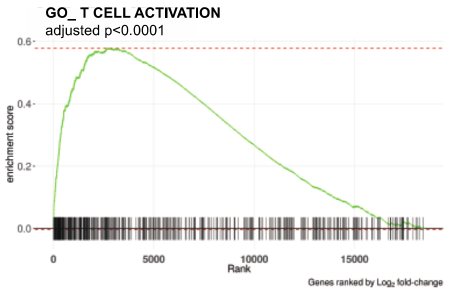

C **D**

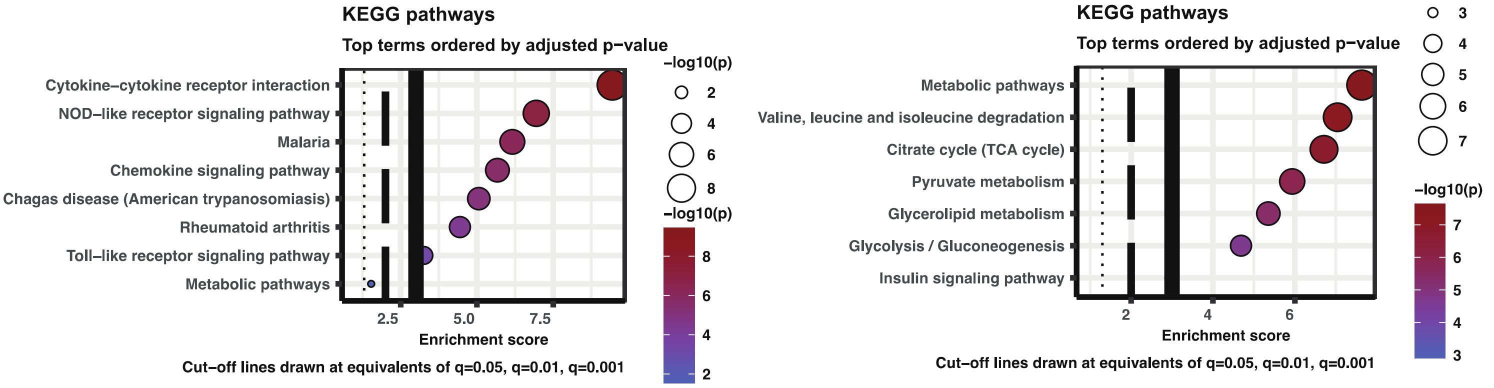

Supplementary figure 5. Transcriptomic analysis of EAT in lean, overweight/obese and T2D patients. (A) Clinical characteristic of the study subjects. (B) GSEA data showing positive enrichment of T cell activation signature in EAT from obese compared to lean patients. (C) KEGG pathway enrichment for overweight/obese vs lean EAT. (D) KEGG pathway enrichment for T2D vs lean EAT. Input genes were those passing adjusted *p* <0.05.

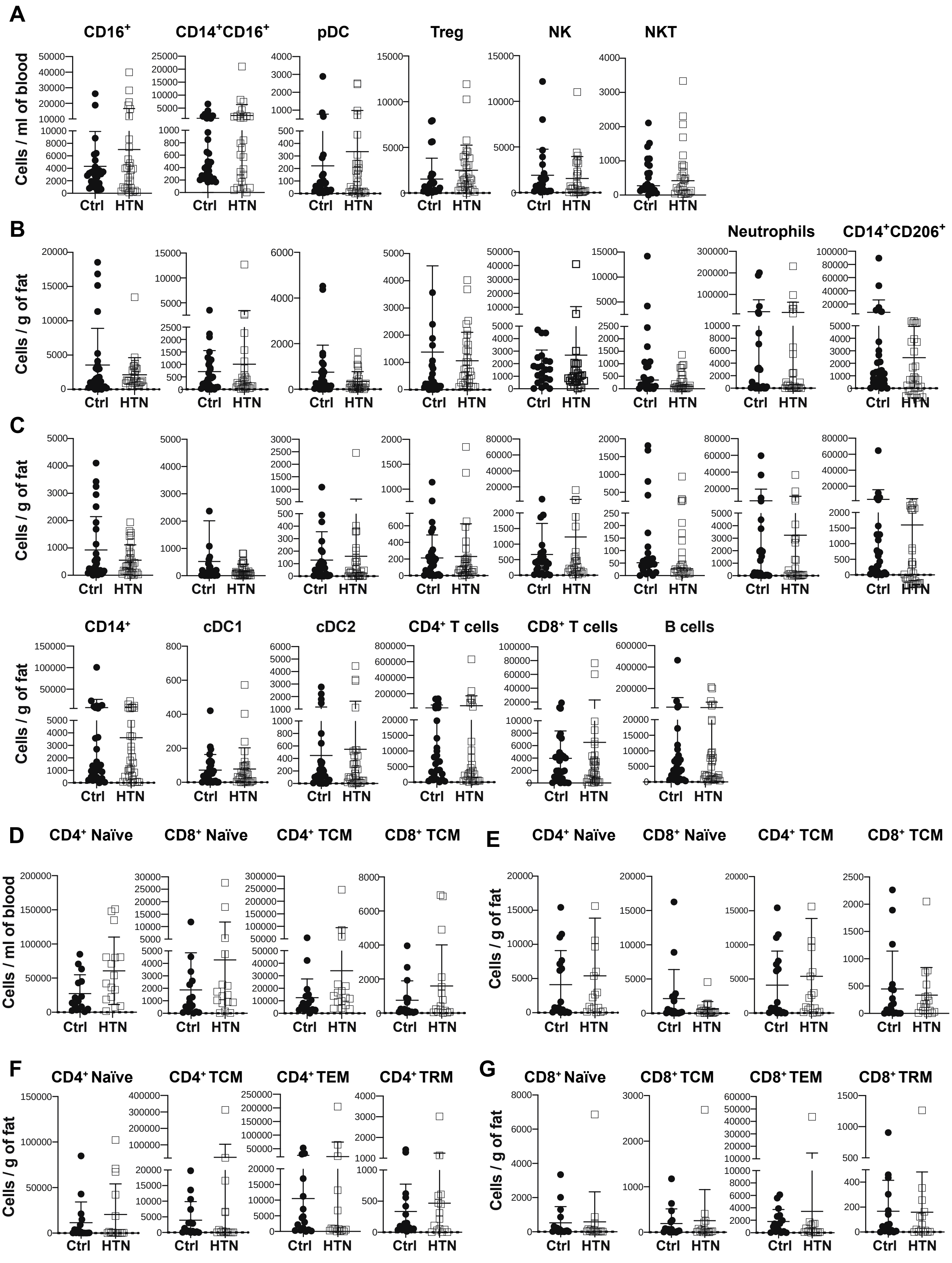

Supplementary figure 6. Absolute numbers of immune cells in blood, EAT and SAT in hypertensive and control patients. Graphs illustrating absolute number of a range of immune cells in hypertensive and control non-hypertensive patients across blood (A), EAT (B), SAT (C). Graphs represent T cell subset absolute numbers in blood (D), EAT (E) and SAT (F-G). Statistical significance was determined by Mann-Whitney test, and data represented as median (n=32 patients/ group).

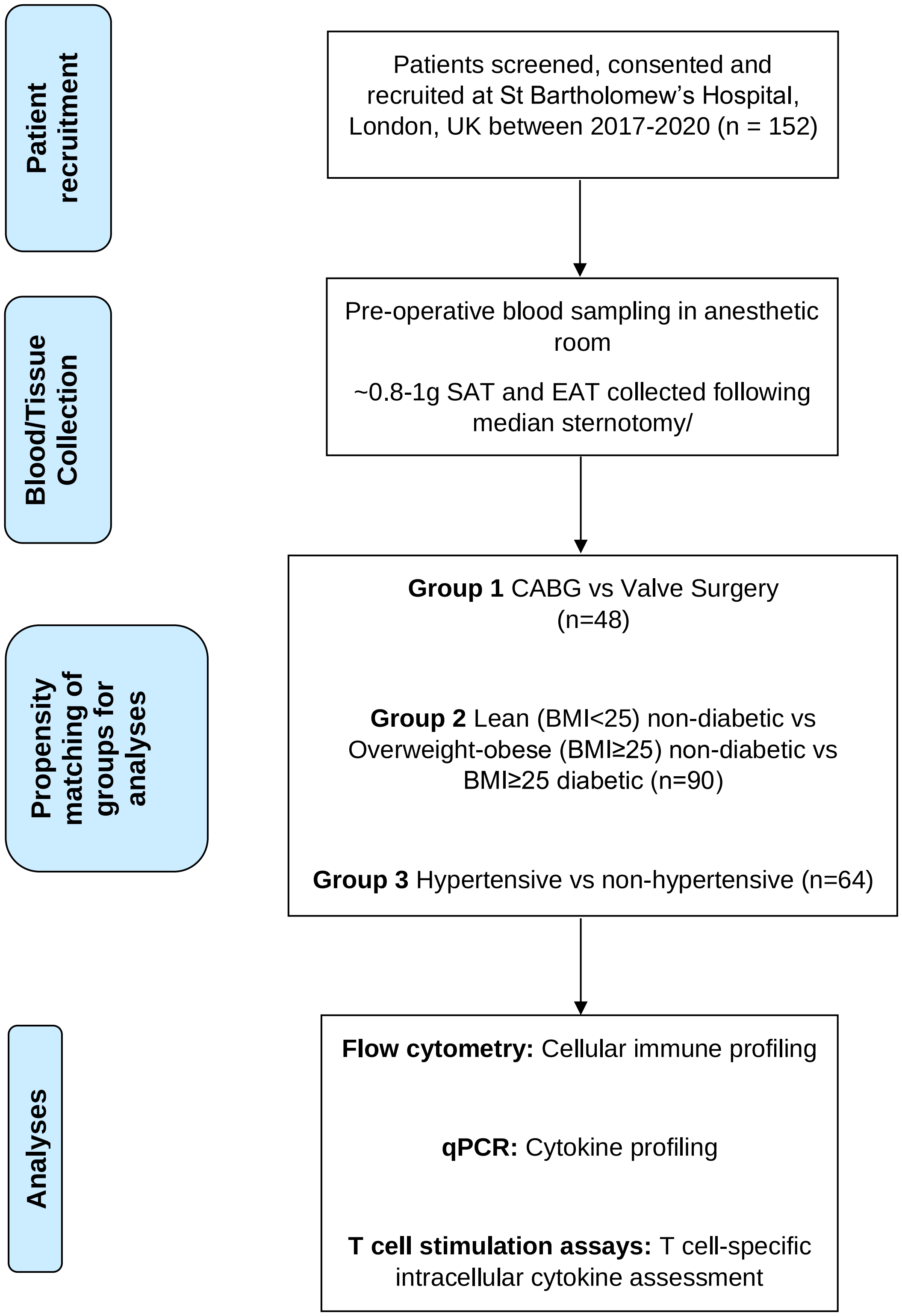

Supplementary Figure 7

Outline of the key steps in patient recruitment, sample collection and sample processing.

Table S1: GSEA enrichment analysis of overweight/obese vs lean EAT

Pathway	padj	NES
GO_MITOCHONDRION	3.6132E-57	-2.3107203
HALLMARK_TNFA_SIGNALING_VIA_NFKB	1.9308E-53	3.50593859
GAO_LARGE_INTESTINE_24W_C11_PANETH_LIKE_CELL	2.3856E-44	3.11234783
GO_REGULATION_OF_IMMUNE_SYSTEM_PROCESS	2.3856E-44	2.23252554
CUI_DEVELOPING_HEART_C8_MACROPHAGE	4.2675E-42	3.12254581
GO_LYMPHOCYTE_ACTIVATION	1.018E-41	2.62476859
GO_CELL_ACTIVATION	1.4317E-41	2.22776502
HAY_BONE_MARROW_ERYTHROBLAST	6.8161E-39	-2.2215223
GO_DEFENSE_RESPONSE	1.0149E-38	2.11106319
GO_MITOCHONDRIAL_MATRIX	1.1873E-37	-2.6774137
GSE14769_UNSTIM_VS_40MIN_LPS_BMDM_DN	1.7564E-37	3.20574288
HU_FETAL_RETINA_MICROGLIA	1.2845E-36	2.84539644
GO_POSITIVE_REGULATION_OF_IMMUNE_SYSTEM_PROCESS	2.0917E-36	2.32462385
GO_ADAPTIVE_IMMUNE_RESPONSE	3.8576E-36	2.79941985
GO_T_CELL_ACTIVATION	7.4542E-36	2.71876369
GSE45365_NK_CELL_VS_CD11B_DC_DN	1.0432E-35	3.18099713
GO_OXIDATION_REDUCTION_PROCESS	6.9555E-35	-2.2552231
BLANCO_MELO_COVID19_SARS_COV_2_INFECTION_CALU3_CELLS_UP	6.4825E-34	2.88989464
GAO_LARGE_INTESTINE_ADULT_CI_MESENCHYMAL_CELLS	2.9155E-33	2.84877296
NAGASHIMA_NRG1_SIGNALING_UP	2.9155E-33	3.16805549
OSWALD_HEMATOPOIETIC_STEM_CELL_IN_COLLAGEN_GEL_UP	1.0465E-32	3.02278276
GO_REGULATION_OF_CELL_ACTIVATION	1.3839E-32	2.53880999
GSE9988_LOW_LPS_VS_CTRL_TREATED_MONOCYTE_UP	1.7515E-32	3.1476328
GO_MITOCHONDRIAL_ENVELOPE	3.236E-32	-2.3168602
GSE26343_UNSTIM_VS_LPS_STIM_NFAT5_KO_MACROPHAGE_DN	1.576E-31	3.08059197
GO_ORGANIC_ACID_METABOLIC_PROCESS	3.2817E-31	-2.1253722
HALLMARK_INFLAMMATORY_RESPONSE	5.6831E-31	3.07542947
GO_REGULATION_OF_IMMUNE_RESPONSE	5.8133E-31	2.27744055
GO_RESPIRATORY_ELECTRON_TRANSPORT_CHAIN	8.592E-21	-2.991854
GO_ATP_METABOLIC_PROCESS	3.5224E-20	-2.5176772
GSE18791_CTRL_VS_NEWCASTLE_VIRUS_DC_4H_DN	7.416E-20	2.59654218
GO_LYMPHOCYTE_ACTIVATION	8.2264E-20	2.09587163
HP_ABNORMALITY_OF_THE_MITOCHONDRION	9.8343E-20	-2.847064
HALLMARK_INFLAMMATORY_RESPONSE	3.5919E-19	2.5387022
GO_ATP_SYNTHESIS_COUPLED_ELECTRON_TRANSPORT	5.1768E-19	-2.9763119
GO_DEFENSE_RESPONSE	5.3484E-19	1.77097284
GSE9988_LPS_VS_VEHICLE_TREATED_MONOCYTE_UP	6.462E-19	2.58554257
GSE2706_UNSTIM_VS_2H_LPS_DC_DN	1.3242E-18	2.54992217
HP_ABNORMAL_BLOOD_GLUCOSE_CONCENTRATION	2.2501E-18	-2.6393139
HALLMARK_INTERFERON_GAMMA_RESPONSE	2.3207E-18	2.5113997
HALLMARK_ALLOGRAFT_REJECTION	2.3929E-18	2.48754546
GO_RESPIRASOME	3.9054E-18	-2.9116465
GO_MITOCHONDRIAL_RESPIRATORY_CHAIN_COMPLEX_ASSEMBLY	3.9694E-18	-2.9069677
HP_ABNORMAL_HOMEOSTASIS	8.444E-21	2.58673224
GO_MONOCARBOXYLIC_ACID_CATABOLIC_PROCESS	5.9345E-18	-2.7757817
HP_HYPERTROPHIC_CARDIOMYOPATHY	8.4011E-18	-2.5415751
HP_ABNORMAL_MYOCARDIUM_MORPHOLOGY	9.2652E-18	-2.2607369
GO_REGULATION_OF_IMMUNE_SYSTEM_PROCESS	1.3817E-17	1.76540132
HP_ABNORMAL_GLUCOSE_HOMEOSTASIS	8.4878E-17	-2.133751
GO_AEROBIC_RESPIRATION	3.8066E-16	-2.9225016

Table S2: GSEA enrichment analysis of T2D vs lean EAT

Pathway	padj	NES
GO_MITOCHONDRION	1.6673E-70	-2.4585369
GO_MITOCHONDRIAL_MATRIX	1.4299E-48	-2.9891375
GO_OXIDATION_REDUCTION_PROCESS	3.0143E-47	-2.4524706
GO_MITOCHONDRIAL_ENVELOPE	6.6112E-45	-2.5920861
MOOTHA_HUMAN_MITODB_6_2002	8.2146E-43	-2.9248399
GO_ORGANELLE_INNER_MEMBRANE	5.9224E-42	-2.8050888
MOOTHA_MITOCHONDRIA	3.9681E-39	-2.8174517
REACTOME_THE_CITRIC_ACID_TCA_CYCLE_AND_RESPIRATORY_ELECTRON_TRANSPORT	6.5374E-39	-3.3203305
GO_MITOCHONDRIAL_PROTEIN_COMPLEX	2.6116E-37	-3.0811668
GO_ORGANIC_ACID_METABOLIC_PROCESS	1.2407E-36	-2.2251642
HALLMARK_OXIDATIVE_PHOSPHORYLATION	5.1294E-36	-3.21005
BURTON_ADIPOGENESIS_6	1.6857E-34	-3.1758217
GO_GENERATION_OF_PRECURSOR_METABOLITES_AND_ENERGY	2.0208E-34	-2.666748
GO_OXIDOREDUCTASE_ACTIVITY	4.0734E-34	-2.4204263
HALLMARK_TNFA_SIGNALING_VIA_NFKB	5.82E-33	2.89606303
HP_ABNORMALITY_OF_ACID_BASE_HOMEOSTASIS	1.3976E-32	-2.852456
HALLMARK_ADIPOGENESIS	2.4573E-28	-3.0147195
GO_CELLULAR_RESPIRATION	6.7767E-27	-3.0237665
GO_ORGANIC_ACID_CATABOLIC_PROCESS	9.4681E-27	-2.7980869
GO_FATTY_ACID_METABOLIC_PROCESS	5.1519E-25	-2.5956071
KEGG_OXIDATIVE_PHOSPHORYLATION	5.5198E-25	-3.0954535
GO_INNER_MITOCHONDRIAL_MEMBRANE_PROTEIN_COMPLEX	2.128E-24	-3.0057297
HP_INCREASED_SERUM_LACTATE	6.1315E-24	-2.9589805
GO_SMALL_MOLECULE_CATABOLIC_PROCESS	1.4956E-23	-2.4178529
REACTOME_RESPIRATORY_ELECTRON_TRANSPORT	1.4956E-23	-3.1065141
GO_ELECTRON_TRANSPORT_CHAIN	1.7887E-23	-2.8907852
GSE9988_LOW_LPS_VS_VEHICLE_TREATED_MONOCYTE_UP	3.6551E-23	2.70148961
GO_ENERGY_DERIVATION_BY_OXIDATION_OF_ORGANIC_COMPOUNDS	4.4333E-23	-2.6907153
GSE2706_UNSTIM_VS_2H_LPS_AND_R848_DC_DN	5.9935E-22	2.67143762
GSE2706_UNSTIM_VS_2H_R848_DC_DN	7.3963E-22	2.65248151
GSE9988_ANTI_TREM1_AND_LPS_VS_CTRL_TREATED_MONOCYTES_UP	2.4552E-21	2.6474521
GSE14769_UNSTIM_VS_60MIN_LPS_BMDM_DN	8.444E-21	2.58673224
GO_RESPIRATORY_ELECTRON_TRANSPORT_CHAIN	8.592E-21	-2.991854
GO_ATP_METABOLIC_PROCESS	3.5224E-20	-2.5176772
GO_LYMPHOCYTE_ACTIVATION	8.2264E-20	2.09587163
HP_ABNORMALITY_OF_THE_MITOCHONDRION	9.8343E-20	-2.847064
HALLMARK_INFLAMMATORY_RESPONSE	3.5919E-19	2.5387022
GO_DEFENSE_RESPONSE	5.3484E-19	1.77097284
GSE9988_LPS_VS_VEHICLE_TREATED_MONOCYTE_UP	6.462E-19	2.58554257
GSE2706_UNSTIM_VS_2H_LPS_DC_DN	1.3242E-18	2.54992217
HALLMARK_INTERFERON_GAMMA_RESPONSE	2.3207E-18	2.5113997
HALLMARK_ALLOGRAFT_REJECTION	2.3929E-18	2.48754546
GO_RESPIRASOME	3.9054E-18	-2.9116465
HP_ABNORMAL_HOMEOSTASIS	4.38E-18	-1.8054708
HP_HYPERTROPHIC_CARDIOMYOPATHY	8.4011E-18	-2.5415751
HP_ABNORMAL_MYOCARDIUM_MORPHOLOGY	9.2652E-18	-2.2607369
GO_REGULATION_OF_IMMUNE_SYSTEM_PROCESS	1.3817E-17	1.76540132
GO_T_CELL_ACTIVATION	1.5212E-17	2.17751458
ZHOU_INFLAMMATORY_RESPONSE_LIVE_UP	2.4537E-17	2.23776561
GO_ADAPTIVE_IMMUNE_RESPONSE	2.6258E-16	2.2037809

Table S3: Antibodies used

Reagent	Source	Catalog Number	Dilution
Anti-human CD197-FITC	Biolegend	353216	1:200
Anti-human CD19-PerCP-Cy5.5	Biolegend	302228	1:200
Anti-human CD45RO-BV421	Biolegend	304224	1:200
Anti-human CD335-BV605	Biolegend	331926	1:200
Anti-human CD45-BV785	Biolegend	304048	1:200
Anti-human CD127-APC	Biolegend	351342	1:500
Anti-human CD8-AF700	Biolegend	300920	1:500
Anti-human CD3-APC/Cy7	Biolegend	300318	1:200
Anti-human CD69-PE	Biolegend	310906	1:200
Anti-human FOXP3-PE CF594	Biolegend	320126	1:200
Anti-human CD4-PE/Cy7	Biolegend	357410	1:200
Anti-human CD303-FITC	Biolegend	354208	1:200
Anti-human CD123-PerCP/Cy5.5	Biolegend	306016	1:200
Anti-human CD206-BV421	Biolegend	321126	1:200
Anti-human CD3-BV605	Biolegend	317322	1:200
Anti-human CD15-BV605	Biolegend	323032	1:200
Anti-human CD19-BV605	Biolegend	302244	1:200
Anti-human CD14-APC	Biolegend	325608	1:200
Anti-human CD16-AF700	Biolegend	302026	1:500
Anti-human CD1c-APC/Cy7	Biolegend	331520	1:200
Anti-human Clec9A-PE	Biolegend	353804	1:200
Anti-human CD1a-PE CF594	Biolegend	300132	1:200
Anti-human CD141-PE/Cy7	Biolegend	344110	1:200
Anti-human IFN-γ-APC	Biolegend	502511	1:200
Anti-human IL-17-APC/Cy7	Biolegend	512319	1:200
Anti-human IL-22-PE	Biolegend	366703	1:200

Table S4: List of primers for RT-PCR

Primer	Forward sequence (5' to 3')	Reverse sequence (5' to 3')
GAPDH	GGCTCATGACCACAGTCCA	CACATTGGGGGTAGGAACAC
IL1	AGCTACGAATCTCCGACCAC	CGTTATCCCATGTGTCGAAGAA
IL6	ACTCACCTCTTCAGAACGAATTG	CCATCTTTGGAAGGTTCAGGTTG
IL10	GACTTTAAGGGTTACCTGGGTTG	TCACATGCGCCTTGATGTCTG
IL12	CTCCTGGACCACCTCAGTTTG	CGGTCATCTGCCGCAAA
IL13	TGCAGCCCTGGAATCCCT	CCCAGCTGAGACCTTGTGC
IL17	AACCGATCCACCTCACCTTG	TCTCTTGCTGGATGGGGACA
IL22	GCTTGACAAGTCCAACTTCCA	GCTCACTCATACTGACTCCGTG
IL23	GCTTCAAAATCCTTCGCAG	TATCTGAGTGCCATCCTTGAG
GMCSF	CATGTGAATGCCATCCAGGA	CAGGCCCACATTCTCTCACTT
CCL17	CTTCAAGGGAGCCATTCCCC	CTCTTGTTGTTGGGGTCCGA
IFNG	TCGTTTTGGGTTCTCTTGGCT	CCTGTTTTAGCTGCTGGCGA
TNFA	GTGACAAGCCTGTAGCCCAT	CAGACTCGGCAAAGTCGAGA
TGFB	CGGCAGCTGTACATTGACTT	TCCAAATGTAGGGGCAGGG
ADIPOQ	AGGAAACCACGACTCAAGGG	CTCCGGTTTCACCGATGTCT
LPL	TCACACACGCAGTCAGTCTC	GAGGTTCTCCAGGTCGTTGG
RETN	TGAAAGCTCTCTGTCTCCTCC	TCCTGGATCCTCTCATTGATGG
